# Useful model organisms, indicators, or both? Ground beetles (Coleoptera, Carabidae) reflecting environmental conditions

**DOI:** 10.3897/zookeys.100.1533

**Published:** 2011-05-20

**Authors:** Matti J. Koivula

**Affiliations:** Finnish Forest Research Institute, Vantaa Research Unit, P.O. Box 18, FI-01301 Vantaa, Finland

**Keywords:** abiotic, assessment, bioindicator, biotic, change, conservation, disturbance, dominance, early warning, human impact, keystone, management, richness

## Abstract

Classic studies have successfully linked single-species abundances, life-history traits, assemblage structures and biomass of carabid beetles to past and present, human-caused environmental impacts and variation in ‘natural’ conditions. This evidence has led many to suggest carabids to function as ‘indicators’ − a term that bears multiple meanings. Here, a conservation-oriented definition for an indicator is used, carabid indicator potential from seven views is evaluated, and ways to proceed in indicator research are discussed. (1) Carabid species richness poorly indicates the richness and abundance of other taxa, which underlines the importance of using multiple taxa in environmental assessments. The ability of assemblage indices and specialist or functional-group abundances to reflect rare species and habitats should be examined in detail. (2) Experimental evidence suggests that carabids may potentially serve as keystone indicators. (3) Carabids are sensitive to human-altered abiotic conditions, such as pesticide use in agro-ecosystems and heavy metal contamination of soils. Carabids might thus reflect ecological sustainability and ‘ecosystem health’. (4) Carabid assemblages host abundant species characteristic of particular habitat types or successional stages, which makes them promising dominance indicators. (5) Carabids reflect variation in ‘natural’ conditions, but vegetation and structural features are more commonly adopted as condition indicators. Carabids nevertheless provide yet another, equally accurate, view on the structure of the environment. (6) Carabids may function as early-warning signalers, as suggested by recent studies linking climate and carabid distributions. (7) Carabids reflect natural and human-caused disturbances and management, but the usefulness of these responses for conservation purposes requires further research. In summary, European carabids appear useful model organisms and possibly indicators because they are diverse, taxonomically and ecologically well-known, efficiently reflect biotic and abiotic conditions, are relevant at multiple spatial scales, and are easy to collect in sufficiently large numbers to allow statistical analyses. The assumption that carabid responses would reflect rare environmental conditions or the responses of rare and threatened species ‒ crucial information for conservationists and managers ‒ has not yet been critically evaluated. Even if it holds, the usefulness will be context dependent: species and their populations vary, conditions vary, questions put forward vary, and assessment goals vary.

## Introduction

Indicators, in the most general sense, can refer to anything that have been shown to reflect something apart from their individualistic response. For example, different species reflect habitat types through their associations with particular biotic and abiotic conditions, and a common assumption is that the magnitude and direction of this reflection are not unique to the studied species. For conservationists and environmental managers, i.e., the potential end users of indicators, such general patterns will not suffice. For them, an indicator should permit conclusions regarding particular conditions or biodiversity, which could not otherwise be concluded either without using the indicator or through using easier, cheaper and/or quicker assessment tools. Indeed, Landres et al. (1988) described an indicator as being a taxon or a structure ”…*whose characteristics (e.g., presence or absence, population density, dispersion, reproductive success) are used as an index of attributes too difficult, inconvenient, or expensive to measure for other species or environmental conditions of interest*“. Here I use the term ‘indicator’ following this strict definition unless stated otherwise.

In ecological impact studies carabid beetles are frequently cited as indicators in the vague sense described above, but according to the strict sense they should more often be cited as model or study organisms. A model organism is a (group of) species that is used to examine a particular study question (a hypothesis) under a research programme (sensu Underwood 1997; see also den Boer 2002). For example, the researcher’s general question might be ”Does fungicide spraying affect soil-dwelling animals?” which is then studied using carabids to model a biological response. If you type the words ’carab*‘ and ’indicator*‘ into Scopus you get 172 results, and similarly ISI Web of Science produces 186 results (26 May 2010). Many if not most of these studies have little to do with carabids indicating anything else but themselves, i.e., their individualistic response to treatments of interest, except perhaps trivial issues such as the sampled habitat type. Such ‘watering down’ of terms may lead to misunderstandings among scientists, practitioners and amateurs, including the media, and to an impoverishment of the scientific language.

Here I evaluate the indicator potential of carabid beetles for seven common applications of indicators (Lindenmayer et al. 2000): (1) indicating richness and abundance of taxa other than carabids; (2) functioning as keystone organisms; (3) indicating human-altered abiotic conditions, here pollution; (4) indicating particular environmental conditions through numerical or biomass dominance; (5) reflecting variation in ‘natural’ conditions; (6) acting as early-warning signalers; and (7) indicating disturbances and management. Generally speaking, the basic requirements for the use of indicators are fulfilled by most European carabids: good knowledge exists on (i) conditions to which these species are adapted to; (ii) distributions of the species in a given set of patches; (iii) the species’ responses to environmental variation/alteration; and (iv) variation in the species’ population dynamics (Andersen 1999; Lindenmayer et al. 2000; see "Carabids as model organisms" below).

In this review I ask three questions with a combined European and North American focus.

Which features characterize carabids as potential indicators? In "Carabids as model organisms" I briefly review the current state of ecological knowledge, information gaps, and methods used in carabid research.

What kinds of indicators might be found among carabids, considering the seven indicator categories above? In other words, what is the evidence for and against using carabids as indicators? In "Evaluation of carabids as indicators" my aim is to summarize key evidence for carabid indicator potential. This Section is intentionally critical, as the use of indicators in conservation should be on an exceptionally solid basis: threatened species or habitats are at stake.

Where, and how, should carabidologists proceed in their search for indicators? In "Identifying and using carabid indicators" I discuss (a) ways to incorporate carabids into routine environmental assessments, (b) issues about carrying out research searching for indicators, and (c) where to find new areas in the ongoing indicator hunt.

## Carabids as model organisms

Prerequisites for being good model organisms and also potential indicators include vast knowledge on carabid taxonomy and ecology, as well as ease of collecting, but these hold mostly only for north-temperate regions (e.g., New 1998). Carabids are taxonomically well known, with relatively stable systematics, and their ecology has been widely studied (Lövei and Sunderland 1996). Variation in carabid morphology, life-history strategies and abiotic and biotic requirements are also extensively documented. We know, for example, many species that are specialized to certain moisture, temperature and shadiness conditions (Rainio and Niemelä 2003; Niemelä et al. 2007). Carabids are also widely distributed, from the arctic and alpine tundra to seashores, deserts and tropical rainforests, and they can be common in these environments (Lövei and Sunderland 1996). However, knowledge about basic life-history parameters appears limited to a few well-studied species. These parameters include birth and death rates, population age structure and growth rate, resource allocation between reproduction and growth, and the causes and magnitude of variation in these. Such parameters are not only interesting but may appear crucial for indicator use (see "Identifying and using carabid indicators").

The reasons for particular distributions, local abundances or behavioral responses of carabids are generally well understood. Carabids are influenced by temperature, moisture and shade (Thiele 1977), food quality and abundance (Lenski 1984; Van Dijk 1994; Bilde and Toft 1998; Bilde et al. 2000; Bohan et al. 2001), habitat structure as reflected by the vegetation (Rykken et al. 1997; Siemann et al. 1998; Brose 2003; Koivula et al. 1999; 2003; Taboada et al. 2008), and substrate salts, sugars and acidity (Merivee et al. 2001, 2004, 2006; Milius et al. 2006). Moreover, seasonal and life-history fluctuations strongly affect observed abundances and distributions (Thiele 1977; Lindroth 1985, 1986; Lövei and Sunderland 1996). Of largely unknown − though often suggested − importance are intra- and interspecific interactions, of which competition has usually had minor effects (Loreau 1990; Niemelä and Spence 1991; Niemelä 1993a; Currie et al. 1996; Zetto Brandmayr et al. 2004).

In ecological research, both landscape and smaller scales appear relevant for carabids, although the former usually requires extensive sampling. Carabids are not always considered relevant at spatial scales larger than a few hectares (e.g., Pearce and Venier 2006). This view relies on the idea of local populations or ‘home ranges’ of carabids (e.g., den Boer 1990a; Gaston and Blackburn 1996; Charrier et al. 1997). However, carabids predictably respond to landscape- (here, areas larger than 50 ha) and even continent-level phenomena (e.g., Hengeveld 1987; Kotze and O’Hara 2003; Kotze et al. 2003). For example, carabids reflected isolation in southern Finnish farmlands (Kinnunen et al. 1996), and responded to patch size and matrix type in an urban landscape in Belgium (Gaublomme et al. 2008). The structural heterogeneity of landscapes had variable impacts on different trophic groups of carabids in Germany (Purtauf et al. 2005). Moreover, carabid assemblages gradually changed across a forest/farmland gradient in Scotland (Vanbergen et al. 2005), and in Canadian post-fire forests, logging variably affected carabids at the stand level but strongly and predictably at the landscape scale (Koivula and Spence 2006).

Most field studies on carabids have used pitfall traps, which is an easy and cheap method to collect sufficiently large samples to allow statistical analysis, by acknowledging that the catch indicates species-specific ‘activity density’ rather than true relative abundance (Greenslade 1964). The dominance of one method over others introduces a knowledge bias. New insights would be achieved by more often applying other collecting methods, such as capture-mark-recapture techniques, trapping and measuring live beetles, window trapping, tree-canopy pesticide spraying, hand collecting, and soil sampling to collect larvae (Sutherland 1996).

The carabid beetle literature reflects a wide spectrum of approaches to study ecological questions. Papers on single species, total abundance and species richness are common. If the numbers of collected individuals are small, or if generalizations are required, carabids are often divided into functional groups to test the hypotheses put forward. These groups include seasonal abundance peak, reproduction period, diurnal activity, body size, wing morphology (e.g., brachypterous/wing-dimorphic/long-winged/flying), food preferences (e.g., predator/omnivore/plant-eater/specialist), associations with habitat openness (e.g., closed tree canopy or extensive vegetation cover/generalist/open phase) and moisture preferences (e.g., dry/moist/wet). Clearly, species divisions into these groups involve subjectivity, because many categories were originally continuous variables, and may be poorly known even in regions with a long research tradition. Flight capability in carabids in Northern and Central Europe is a good example of such knowledge gaps (Niemelä et al. 2007). Morphospecies or higher-than-species level approaches are rarely applied by carabidologists, because different species within a genus are ecologically different and may consequently respond differently to the environment (Koivula et al. 2006; Langor and Spence 2006).

Various diversity indices have been used on the carabid catch. These include, for example, rarefaction (Sanders 1968) and the Shannon-Wiener and Simpson indices (Magurran 2003; Tóthmérész and Magura 2005a). However, diversity indices may perform inconsistently (O’Hara 2005) and therefore should not be used as a sole justification of indicator functioning. Another obstacle is that diversity measures based on pitfall-trap data are problematic because the samples are biased toward actively moving, large-sized species (e.g., Morrill et al. 1990; Lang 2000). As such, these samples may have little to do with true assemblage composition and structure. The relationship between trap samples and true assemblages is poorly understood due to the difficulty in reliably determining the latter.

Recent approaches to describe carabid assemblage structure include Mean Individual Biomass (Szyszko et al. 2000; see "Dominance indicators"), affinity indices (Allegro and Sciaky 2003; Tóthmérész and Magura 2005b) and indicator value calculations (IndVal; Dufrêne and Legendre 1997). Affinity indices aim at removing the effect of differences in species abundances among compared habitat types while simultaneously accounting for the species’ habitat specificity (Magura et al. 2006a). The IndVal approach uses data collected from habitat types of interest, and identifies species characteristic of particular habitat types based on their abundances and presences/absences among all samples (Dufrêne and Legendre 1997).

## Evaluation of carabids as indicators

### Taxon indicators

The presence of a taxon indicator reflects the presence of a set of other species, and its absence indicates the absence of the entire set of species (Slobodkin et al. 1980; Lindenmayer et al. 2000). The underlying assumption thus is that the presence of a limited subset of all species would indicate the presence of the complete set. As everything cannot be measured this approach may sound appealing, but evidence of carabids as taxon indicators is poor. Weak richness correlations with carabids have been demonstrated for spiders (Rushton et al. 1989; Niemelä et al. 1996) and some other invertebrate taxa (Duelli and Obrist 1998; Niemelä and Baur 1998). Barbaro et al. (2005) found that the same structural features of forests predicted bird, spider and carabid richness in France. The utility of richness indicators becomes even more challenging at larger spatial scales, where richness correlations appear to be a biogeographic rule. Species richness of different taxa often correlate because of the general tendency of richness to increase toward the equator (Begon et al. 1996); for a national-scale invertebrate example, see Väisänen and Heliövaara (1994).

The taxon indicator potential of carabid beetles has not yet been subject to a severe test (sensu Mayo 1997), but such tests do exist for other taxa. Jonsson and Jonsell (1999) showed that stand structure and the richness of taxa bearing high conservation relevance (lichens, plants, wood-rotting fungi and bryophytes) appeared to be poor *a priori* indicators of each other in Swedish boreal forests. Likewise, Similä et al. (2006) found that structural characteristics and plant richness somewhat reflected the richness of some invertebrate groups, but beetles very poorly reflected the richness of other taxa in Finnish boreal forests. Moreover, Sætersdal et al. (2005) showed that the degree of overlap in richness among six ecological groups, consisting of polypores, bryophytes and lichens, varied considerably from site to site in Norwegian coniferous forests. While discouraging, these results highlight the importance of using multiple taxa in environmental assessments (cf. Taylor and Doran 2001; Duelli and Obrist 2003; Paillet et al. 2009) and the absurdity of the idea of the existence of a single ‘biodiversity indicator’.

Conservationists and managers generally agree in that protecting species diversity is a priority at global and national scales. At smaller spatial scales, however, richness may appear a misleading conservation measure without considering species identities. For example, Koivula and Spence (2006) showed that, in recently burned Canadian forests, logging increased the total richness of carabids due to the colonization of generalist open-area associated species. But simultaneously most closed-forest species decreased in abundance, the most drastic case being the over tenfold decrease of *Calosoma frigidum*, a tree-canopy caterpillar hunter (Larochelle and Larivière 2003). So, at the operational scale of individual forest stands, should the forest manager adopt the message obtained from total richness or that from species requiring closed forests?

### Keystone indicators

A keystone indicator is a species, a group of species, or a structure that affects its environment and therefore other species disproportionately strongly relative to its abundance (Mills et al. 1993). The lack of a keystone indicator would thus lead to major changes in some other species’ occurrence, abundance and/or distribution. A classic example from forested environments is the woodpecker fauna (Virkkala 2006). These birds produce nesting sites for secondary cavity-nesters, are important vectors for wood-rotting fungi, and may even regulate bark beetle infestations, thus bearing economic importance (Fayt et al. 2004). Carabids have intrinsic biodiversity value and unknown future potential, and they can also be considered invaluable on an ethical basis, but can they serve as keystone indicators?

Evidence on the importance of carabids comes from agro-ecosystems, greenhouses and laboratories. Under laboratory conditions carabids forage efficiently on slugs and eggs, pupae, larvae and adults of pest insects (Kromp 1999). In the field, carabids indeed prey on pest invertebrates, such as slugs, aphids and mites (e.g., Allen 1979; Edwards et al. 1979; Hengeveld 1980a, 1980b; Luff 1987; Sopp et al. 1992; Bohan et al. 2001). Menalled et al. (1999) manipulated onion fly (*Delia antiqua*) pupae using exclosures in corn fields and found a positive relationship between carabid abundance and pupal death rates. But can the rates of foraging in the field be ecologically and/or economically important?

Hance (1987) used 1 m2 enclosures with sugar beet and natural densities of aphids feeding on these plants, and released 0–30 individuals of *Anchomenus dorsale* and *Asaphidion flavipes* into these enclosures. Such densities (up to 30 ind.m-2) are common in the field (Lövei and Sunderland 1996). In enclosures without carabids, the density of aphids increased exponentially. At intermediate carabid densities, the aphid increase was delayed, and at high carabid densities the aphids often did not increase at all. It is easy to argue that this is ecologically and economically important, contrary to some ‘statistically significant’ 20–30% abundance changes. While this experiment can be criticized for using unrealistic, closed miniature systems, it shows that carabids have the potential for being economically important.

Carabids thus have the potential, but lack field-based evidence, for truly functioning as keystone indicators. Are carabids necessary for ecosystem functioning, and even if they are, could other taxa replace them if they are removed from an ecosystem? Currently there are no answers to these questions, but in many ecosystems carabids are accompanied by other abundant generalist invertebrates, such as ants, staphylinid beetles and spiders (Turnbull 1973; Bohac 1999). Carabids are, on average, larger than these three, which suggests a higher trophic level and per capita effect on, for example, crop-pest invertebrates. On the other hand, carabids are often vastly outnumbered or even excluded by *Formica* wood ants in Fennoscandian boreal forests (e.g., Koivula et al. 1999).

### Pollution indicators

Pollution indicators reflect human-altered abiotic conditions in the soil, water and the air (Spellerberg 1994). Urban ecological studies might be considered in this category, with the combined role of e.g. pollutants, soil compaction and the ‘heat island’ effect (Forman 2008; Marzluff et al. 2008). Pollution affects humans directly, and as such has been studied widely for several decades using several taxa, of which lichens may be the most famous (Lindenmayer et al. 2000). Other pollution indicators, too, have been proposed but not without problems. For example, the mollusc *Velesunio ambiguus* was long considered an excellent indicator of heavy metals in aquatic systems until it appeared that this species’ uptake of metals did not reflect the extent of pollution (Lindenmayer et al. 2000).

Carabids have been commonly studied to evaluate the ecological effects of industry emissions and agriculture chemicals. The below examples demonstrate the potential for carabids to also act as indicators of ecologically sustainable farming, environmental recovery and ‘ecosystem health’. The utility of carabids as indicators in these cases relies on the inadequately tested assumption that other, often more severely threatened, taxa similarly respond to these pollutants and chemicals. This issue concerns the other indicator categories as well.

Several case studies all suggest that heavy metals in the soil significantly and negatively affect carabids (e.g., Ermakov 2004; Gongalsky et al. 2004; Belskaya and Zinoviev 2007). Moreover, cadmium and zink affect the growth and body caloric value of *Poecilus cupreus* individuals (Maryański et al. 2002). Carabids have also been used to assess the recovery of ecosystems after pollution events (e.g., Schwerk et al. 2006; Cárdenas and Hidalgo 2007).

In agro-ecosystems, pesticide and fertilizer impacts on carabids have been studied (e.g., Dritschilo and Erwin 1982; Basedow 1990; Kromp 1990; Larsen et al. 1996; Bourassa et al. 2008). Carabids respond negatively to dimethoate (commonly-used pesticide) sprayings but their numbers may recover within a few weeks (Huusela-Veistola 1996). Fertilizer and herbicide impacts have often been minor, but may affect carabids indirectly through changes in the vegetation (Kromp 1999).

Also cumulative impacts may appear common. For example, the intensity of carabid response to pollutants and chemicals depends on additional stressors, such as food scarcity and chemicals. Stone et al. (2001) studied adults of *Pterostichus oblongopunctatus* at a chronically polluted mining area in Poland. They collected individuals at sites with different levels of soil metals and subjected these beetles to food shortages and an insecticide (dimethoate) in the laboratory. Carabid death rates, caused by these stressors, were higher the more severely the collecting site had been contaminated by metals. To determine whether these responses were genetically based or resulted directly from soil contamination, Lagisz and Laskowski (2007) collected additional individuals at Stone et al.‘s (2001) sites, and reared a second generation in the laboratory. These laboratory specimens were subjected to food shortages and the same insecticide, and results showed that the collecting site of the parent individuals had no effect on death rates of the second generation. Thus, the interaction was not genetically based in this case.

Recent advances in agro-ecosystems concern gene-manipulated (GM) or transgenic plants that can be considered ‘genetic pollutants’, as evidenced by the hybridization of native and GM corn in Mexico (Quist and Chapela 2000). GM techniques have been rapidly adopted into agriculture to increase the crop plants’ pest and disease tolerance, yield and/or nutritional value, but manipulating the genetic material of these plants is suspected to lead to unwanted consequences (e.g., Dunwell 1999). For example, the use of GM plants might directly or indirectly affect non-target organisms, including carabids. Non-target invertebrates were generally little affected by GM corn and cotton, as compared with non-transgenic versions of these plants, but were more affected by the use of pesticides (Marvier et al. 2007). Similarly, GM crops had a minor effect on adult carabids locally (Lopez et al. 2005; Szekeres et al. 2006; Floate et al. 2007). However, Waltz (2009) summarized the effects of GM crops on insects and reported drastic effects on, e.g., butterfly larval death rates. Hence, experiments on the larval development of seed-eating carabids in GM and conventional crop fields would significantly contribute to this area of research.

### Dominance indicators

Dominance indicators make up much of the total biomass or the number of individuals in an area of interest (Lindenmayer et al. 2000) and predict particular ecosystems or assemblages. For example, certain tree species form much of the biomass and broadly reflect habitat type in forests. Similarly, carabid dominance indicators should reflect particular habitat types, degrees of disturbance and ecosystem recovery, hot-spots of rare species or particular habitat types of conservation interest. The use of carabids in this sense has faced certain difficulties that might be overcome.

Invertebrates are seldom used in environmental assessments because of the high expertise required (Andersen 1999; but see Andersen and Majer 2004). While strongly advocated here (see "Carabids as model organisms"), species-level approaches usually require considerable investments of expertise, time and money into education, sampling and analysis (Langor and Spence 2006). Hence, in rapid biodiversity assessments (e.g., Ward and Larivière 2004), numerical or biomass dominance might be alternative options.

Niemelä (1993b) showed that boreal-forest carabid assemblages consist of a few abundant (easily identifiable) and several scarce (often more difficult to identify) species. In these forests, early successional phases can be numerically dominated by *Pterostichus niger*, while closed phases are often dominated by *Calathus micropterus* (e.g., Koivula et al. 2002). However, as these species are generalists of forest succession (Niemelä et al. 2007) and occur in many forest types (Lindroth 1985, 1986), their presence may not indicate aspects useful for conservation or management.

Carabid body size has been linked to certain ecological processes, such as urbanization and succession (e.g., Magura et al. 2006b). The Mean Individual Biomass (MIB) approach requires only sampling, counting, weighing and using a simple equation developed by Szyszko et al. (2000). MIB is predicted to increase along gradual successional changes in vegetation that subsequently alters the carabid fauna, from smaller open-habitat (*Amara*, *Bembidion*, etc.) to larger closed-forest (*Carabus*, *Cychrus*, etc.) species (Szyszko et al. 2000). An increase in MIB should thus indicate conditions approaching late successional stages.

MIB is advocated as an easy tool for policy makers to assess the state of the environment. The method assumes a linear relationship between MIB and time since disturbance, which seems to hold through early successional phases, during which the carabid fauna changes rapidly (Szyszko et al. 2000; Koivula et al. 2002). However, at least in boreal spruce forests the carabid assemblage structure − and consequently MIB − changes little between 30 and 100 years following clear-cutting (Koivula et al. 2002; M. Koivula unpubl.), suggesting a plateau in the trend. For forests older than 100 years, MIB might even decrease, as these ‘old growth’ phases are characterized by disturbances that create new habitat for species associated with tree-canopy openness. In forests, these disturbances include falls and deaths of single or small groups of trees (Esseen et al. 1997; Bouget 2005; Skłodowski 2007). The ‘behavior’ of MIB warrants further research before applying it in conservation and management, but it may already have potential in landscape-level assessments.

### Environmental indicators

An environmental indicator reliably reflects particular environmental conditions in soil quality, moisture, flooding regime, and so on (Klinka et al. 1989). Plants in particular have been widely used as indicators of e.g. soil quality, water levels, habitat types and, based on Christen C. Raunkiær’s growth-form descriptions, biomes (Begon et al. 1996). Although carabids also have the potential to reflect soils, wetness and habitat-type variation, they cannot currently compete with plants as environmental indicators for these factors.

Carabids efficiently reflect environmental variation, and bear indicator potential at various spatial scales. For example, variation in soil conditions within a few meters affected farmland carabid diversity in England (Sanderson et al. 1995). At larger scales, distinctive carabid assemblages are found at lake, river and sea shores, bogs and mires to very dry habitats (e.g., Lindroth 1961–1969, 1985, 1986; Larochelle and Larivière 2003), temporary wetland pools (e.g., Uetz et al. 1979; Brose 2003; Gerisch et al. 2006; Follner and Henle 2006) and in dry and sandy heathlands and grasslands (e.g., Vermeulen 1993; Magura and Ködöböcz 2006).

Carabids have occasionally been used as environmental indicators. Eyre and Luff (1990) attempted to classify European grassland habitats using carabids. They sampled 638 sites in Northern and Central Europe and distinguished 17 grassland types that were often shared among several countries. Likewise, Eyre et al. (1996) and Eyre and Luff (2002) classified riverside habitats using carabids. They distinguished several site groups, each with distinctive structural characteristics and associated carabid species. The value of carabids here is that they produced different but equally correct site classifications as compared with traditional, vegetation-based approaches.

The above examples concern relatively stable conditions, but carabids might be useful also in assessing *changes* in conditions (see "Early warning indicators") due to the ability of many species to disperse by flying. For example, the first colonizers appear within a few weeks or months following forest fires (e.g., Burakowski 1986; Koivula et al. 2006). Fragmentation provides a particularly promising framework in this sense. Due to fragmentation, similar-looking habitat patches vary in size and isolation, which might be reflected by the proportion of winged and wingless individuals. West European carabids have been classified based on their habitat affinity and ability to disperse, and these traits predict population extinctions and colonizations in fragmented heathland networks quite well (Turin and Heijerman 1988; Turin and den Boer 1988; Desender and Turin 1989; den Boer 1990b; de Vries et al. 1996).

### Early warning indicators

Early-warning signalers are extremely sensitive to changing environmental conditions (Lindenmayer et al. 2000). Conditions of interest are often at large spatial scales, such as fire, climate, or the spread of urban areas. Species in this category are often referred to as true ’bio-indicators’. What is the evidence for carabid functioning as early warning indicators?

Many studies have documented changes in carabid assemblages due to drastic habitat alterations caused by forestry, wildfire, grazing, fertilization, fragmentation and so on (for reviews, see Luff 1987; Lövei and Sunderland 1996; Kromp 1999; Niemelä et al. 2007). For example, carabid responses to clear-cut harvesting are usually detectable within 1–3 years (e.g., Niemelä et al. 1993; Koivula 2002a). Of course, these responses may not always be clear and other taxa may more readily respond to changes in habitat quality (e.g., Matveinen-Huju et al. 2009), emphasizing context specificity of indicators. Another problem is that in many of these studies carabids did not truly indicate condition alterations before they became visually obvious, thus did not act as early warning indicators.

Climate change has dominated headlines for the past 10–15 years. High-impact journals have eagerly printed research on the climate responses of butterflies, frogs and birds (e.g., Parmesan et al. 1999; Pounds et al. 1999; Cotton 2003; Hüppop and Hüppop 2003). Carabids, too, reflect changes in climatic conditions but the rate of change in their distributions is largely unknown. Butterfield (1996) showed that carabid samples collected at 450 and >800 m a.s.l. were different, and Ashworth (1996) found fossil remains to indicate that the carabid fauna 10 000 years ago was different from the current fauna at the same sites. Preliminary results of two European studies suggest that carabids have moved tens of meters in altitude in the past 10–20 years (Assmann 2009; Pizzolotto 2009), coinciding with the general predictions of climate warming (Parry et al. 2007). Climate change possibly also interacts with other environmental factors, such as those associated with urbanization. For example, Bednarska and Laskowski (2009) showed that the death rate of larvae of *Pterostichus oblongopunctatus* was significantly affected by a combination of temperature and soil nickel content.

### Disturbance and management indicators

Disturbance indicators reflect natural and human-caused disturbances (Milledge et al. 1991), whereas management indicators reflect human efforts in decreasing the biological impact of these disturbances (e.g., Günther and Assmann 2005). Again their usefulness relies on the assumption that what is detected by the indicator is similarly affecting other, often threatened, taxa. In forestry, for example, several taxa respond to cutting of live trees in similar ways (see Barbaro et al. 2005): openness-associated species increase and closed-canopy specialists decrease, as have been shown for boreal ground-dwelling carabids (Niemelä et al. 1993; Koivula 2002a), plants (Jalonen and Vanha-Majamaa 2001) and birds (Koivula and Schmiegelow 2007). Although the indicator functioning clearly holds at this general level, whether these taxa function as indicators of each other in terms of spatial overlap (their predictive accuracy) is yet to be evaluated. Additional problems are many: for example, rare and threatened species may also respond to factors other than live-tree removal, such as the retention of snags or single live and dead trees (e.g., Kaila et al. 1997; Martikainen 2001). Results on epigaeic fauna sampled using pitfall traps may not necessarily apply to species associated with dead wood (but see Work et al. 2008) or canopy dwellers.

Structure-based disturbance (and environmental) indicators are commonly used for practical purposes. For example, in Fennoscandian and British forests, the quality and quantity of live and dead trees, certain biotopes, and signs of forestry are used together to indicate forests of high conservation priority, such as old-growth forests (Hallman et al. 1996; Angelstam 1997; Humphrey and Watts 2004; Hakalisto et al. 2008). These variables reflect rare habitat types, which are crucial for threatened forest species (e.g., Rassi et al. 2001; Gärdenfors 2005). Preliminary results on threatened polypores in Southern Finnish forests suggest that these structure-based indicators allow an efficient identification of stands of high conservation value (Juha Siitonen and Reijo Penttilä, Finnish Forest Research Institute, unpubl.). Could boreal forest carabids reflect variation relevant for conservationists and managers?

Carabid sensitivity to environmental variation suggests good potential here. The early phases of forest secondary succession are characterized by a different set of species than are the later phases with a closed tree canopy (e.g., Niemelä et al. 1993, 2007; Spence et al. 1996; Beaudry et al. 1997; Abildsnes and Tømmerås 2000). Carabids also respond differently to different logging regimes. Compared to unharvested stands, thinning (10–30% removal of trees) affects carabids only marginally, cutting small gaps (diameter 30–50 m) has variable impact, and clear-cutting causes open-area and succession-generalist species to increase and closed-forest carabids to decrease (e.g., Koivula 2002a, 2002b; Vance and Nol 2003; Work et al. 2004). Suggested closed-forest specialists are many but views may change with time: Halme and Niemelä (1993) proposed *Carabus glabratus*, *Carabus violaceus* and *Cychrus caraboides* to be such, but fifteen years later only the latter remained in this list (Niemelä et al. 2007). The reason is not rapid evolution but an accumulation of ecological knowledge. Finnish spruce-forest carabid assemblages change remarkably during the first 20–30 years following clear-cutting, but not much after that, as samples from 60- and 100-year old forests are relatively similar (Koivula et al. 2002; M. Koivula, unpubl.). These carabids thus reflect canopy closure for sure, but the usefulness of this information in conservation and management is obviously low.

Perhaps particular boreal species would be useful indicators? *Platynus mannerheimii* is a suggested old-growth forest spruce-mire specialist (Lindroth 1986; Niemelä et al. 1987, 1993; Gärdenfors 2005; Paquin 2008). However, this species has also been found in 60-year old regenerating stands (Koivula et al. 2002) and along roadsides (Koivula 2005), indicating more flexibility in habitat use and/or dispersal ability than previously thought. Even if this species reliably indicates mire patches worthy of special attention in forestry, such sites are easier identified using structural characteristics and vegetation (Hakalisto et al. 2008). At first glance Finnish forest carabids may not appear specialized enough for conservation and management purposes. This view may appear premature, however: attention could also be paid to the abundances/proportions of potential indicators rather than solely to their presence/absence (see "Identifying and using carabid indicators").

The message here is not that carabids would generally be useless management indicators, but rather that in the particular context of boreal managed forests, with the present state of knowledge, they are not useful. Indicator usefulness should be evaluated separately, depending on the context, for other habitat types, management questions or geographic areas and so on. In Western and Eastern Europe, the carabid fauna of ancient woodlands (forests covered by mature trees continuously at least since the end of the 18th century) differs from that of managed forests (Assmann 1999; Magura et al. 2002, 2003; Desender 2005; Skłodowski 2006; see also Davies and Margules 1998), and *Carabus variolosus* may indicate conditions characteristic for swamps and brooks of ancient woodlands (Matern et al. 2008). Geographic and/or habitat-type differences in carabid responses are common. For example, across grassland/closed-forest edges in Hungary, the grasslands, edges and forests hosted distinctive carabid assemblages (Magura et al. 2001; Lövei et al. 2006), but across clear-cut/closed-forest edges in Finland, edges differed from clear-cuts but were similar to the forest in this respect (Heliölä et al. 2001). A given species may also occur in different habitats in different regions (see discussion in Koivula et al. 2006).

## Identifying and using carabid indicators

### Sketching a road map for detecting useful indicators

Collecting data easily and cheaply, and then using these data to generalize about entities worth special attention, is an appealing idea. Indicators are more and more commonly applied in conservation and management through years of research (Meffe and Carroll 1997). Examples include the uses of habitat structure for identifying forests of high conservation value (Hakalisto et al. 2008), vegetation for identifying habitat types (Klinka et al. 1989) and ants for assessing effects of land management (Andersen and Majer 2004). Carabids have not yet been commonly incorporated into assessments of environmental change, biomonitoring programs, or protocols for identifying sites of high conservation value. Carabids are nevertheless promising candidates for these purposes. Instead of investing resources in finding completely new indicators, we should (1) identify a selection of easily-sampled and ecologically well-known taxa that cover multiple dimensions of biodiversity, and (2) critically evaluate their indicator functioning (Langor and Spence 2006).

Carabids have seldom, if ever, been used or even considered as indicators by conservationists and managers. This may result from (a) carabids being less appealing and charismatic than many hairy/feathered and large-eyed vertebrates; (b) carabids being inconspicuous and therefore easily overlooked by an untrained person; (c) the idea that protecting larger species with larger home ranges would simultaneously secure the well-being of smaller species (the umbrella species concept; see Simberloff 1998); and (d) carabids being uninteresting generalists that are laborious to collect and difficult to identify compared to, e.g., vegetation characteristics of a focal patch. This state of affairs can be changed, but it requires advertising campaigns (such as the Jakhalzen show about the XIV ECM on Dutch television on the 2nd of October 2009) and detecting a ‘niche’ for carabid use as indicators. For the latter goal it is important to increase knowledge about biodiversity covariation, to develop large-scale sampling networks, to develop and test easy-to-use approaches, and to initiate databases for life-history and indicator-concept information about carabids, including data on taxon overlap.

There is an urgent need for clarifying the abundance and response relationship between carabids and other taxa before using carabids in environmental assessments. Correlations between focal taxa are not enough for judging the adequacy of the proposed indicator − spatial and temporal overlapping, predictive power and error estimates must also be evaluated (see "Indicator hunt: common sense revisited").

Indicators need not be used to identify the obvious: for example, the conservationist does not need carabids to decide whether a clear-cut forest has experienced a considerable environmental change. More useful information in this example would be, e.g., how precisely species, functional groups and/or relative abundances of carabids reflect rare species. But conservationists and managers very often sample only at the focal site to decide whether the site is worth protecting. For such purposes, the assessment is difficult to do by using abundance and compositional data, because the composition is never stable due to factors of interest mixing with e.g. species-specific temporal variation. This difficulty might be overcome by defining limits for ‘natural’ variation in the indicator’s abundance or proportion, which requires detailed information about population dynamics and thus long-term sampling in varying conditions (see "Carabids as model organisms").

The accumulation of knowledge may change how we see species, and thus relying on a single study may be a poor strategy. This is particularly important in selecting indicators, because the use of an inappropriate indicator may cause severe conservation and economic harm (Baker and Schonewald-Cox 1986). Species classifications based on only one or a few studies to derive habitat associations perpetuate a view that any species is a specialist (of ‘open’ or ‘closed’ canopy, for instance). As "Disturbance and management indicators" showed, this issue is not that straightforward. Carabids often occur across wide sections rather than at strictly delimited points of the multi-dimensional environmental space, and case studies seldom capture this pattern. Commonly-shared frameworks to keep track of the knowledge about habitat associations and other life-history variables, as ecological studies accumulate, are lacking but would be useful for indicator purposes.

An extensive use of assemblage composition as indicators may require reference sites. Concretely, this could mean a carabid equivalent of the Finnish National Forest Inventory (www.metla.fi/ohjelma/vmi/info-en.htm): a large-scale, long-term, reference sampling network. The first step towards a national protocol might be to establish smaller networks at areas with most critical conservation situation. The often remarkable variation in assemblage composition between adjacent, similar-looking sites, even within a given patch (Niemelä et al. 1992; den Boer 2002), suggests that such networks must be very dense and use high sampling effort. Volunteers could perhaps be used here to ease the work load of professionals. Moreover, the establishment and proper use of such networks involve high sampling-design and taxonomic expertise. Therefore, the development of simple, quick and cheap indicators (such as body-size based) should also be among the priorities. But how to concretely collect data relevant for conservationists looking for useful indicators?

### Indicator hunt: common sense revisited

One of the basic issues is to clarify whether the researcher uses her/his favorite taxon as an indicator or simply as a model organism. To evaluate the indicator potential of carabid beetles, the following tips may be useful.

1. Define *a priori* what you would like (carabids) to indicate, i.e., state an assessment goal (Simberloff 1998; Caro and O’Doherty 1999).

2. Clearly define the aims, methods and appropriate spatial scale *a priori* (Underwood 1997; Duelli and Obrist 2003).

3. Experimentally test the functioning of the potential indicator (Mayo 1997; McGeoch 1998; Caro and O’Doherty 1999; Langor and Spence 2006).

4. Sample long enough, preferably for a number of years, to account for variation in temporal abundance and diversity (Lövei and Sunderland 1996).

5. At each study patch (replicate), sample extensively to cover multiple local populations (den Boer 2002) and within-patch variation.

6. Through analysis and critical interpretation of the data, explicitly state the specific entities and conditions the indicator reflects.

7. Identify and define sources of subjectivity (Landres et al. 1988; Caro and O’Doherty 1999).

8. The validity of the indicator should be evaluated independently.

9. Even if found successful, use the indicator only if other assessment options are unavailable (Landres et al. 1988; Lindenmayer et al. 2000).

Of course, the appropriateness of an indicator can be tested in many ways. There is room for descriptive studies in evaluations of spatial and temporal overlap between taxa, but otherwise experiments are crucial. Comparisons of replicated, unaltered controls with other treatments or collecting multiple samples along environmental continuums may prove useful. Replicate treatments not just samples (Hurlbert 1984). An example may clarify these issues.

Assume you are interested in the impact of a fertilizer on meadow biodiversity, and you would like to study if carabids respond to the added fertilizer as an early warning indicator, i.e., before it can be detected by inventorying plants. You might have a reason for expecting some carabid species to be able to do so (see Merivee et al. 2006). You decide to explore slight differences in assemblage composition using pitfall traps.

The study can be done by sampling, for example, (i) several treated (fertilizer added) and untreated (no fertilizer added; control), randomly-assigned sub-plots within one or a few meadows. Such a protocol would be suitable for detecting small-scale phenomena, such as variation within meadows; (ii) several (say >10) meadows treated with different levels of the fertilizer. This protocol might be fine for assessing threshold conditions by using non-linear regression modeling to evaluate, e.g., if the threshold of abundance change occurs earlier for carabids than for plants; (iii) multiple meadow pairs of which one is treated and the other is not; or (iv) separate, treated and untreated meadows (see, e.g., Underwood 1997).

Assume that you end up using the last-mentioned option. A convincing demonstration of your case would require at least the following.

a. Select meadows that are initially as similar as possible but still distinctive.

b. Establish at least 3–4 treated and 3–4 untreated meadows to be able to calculate means and variances for both. The more meadows the better, as more natural variation will be covered and the more precise the estimate of mean. If possible, sample before and after the addition of the fertilizer to better account for initial variation (Underwood 1992). Concerning your study question, these meadows (not traps in them, irrespective of how they are placed) are your replicates: you are interested in a phenomenon that scales to variation between meadows.

c. Spatially distribute your replicates evenly. They should not form treatment-specific clusters.

d. The replicates should be separate, i.e., unlikely to affect each other ecologically. Sections of different habitat types between your study meadows help convince your colleagues that the meadows are indeed ecologically independent from each other.

e. Synchronize the sampling, i.e., sample at every meadow over the same period.

f. Collect multiple samples from each meadow (see point 5 above).

g. Sample over a period long enough to representatively collect carabids, and also to see if the plant assemblage responds to the treatment. If the plants, or any other taxa other than carabids, do not respond to the treatment, you have failed to find an early warning indicator, whatever your result for carabids. The follow-up may easily take several years to produce useful information.

A lack of proper replication is surprisingly common in ecology, considering the amount of literature on this issue. In the above example, you might have selected only one treated and one untreated meadow and set 10 traps in each, perhaps 15–20 m apart for sample independence (Digweed et al. 1995). But you would then have no replication for the factor of interest, viz. the addition of fertilizer, which operated at the meadow scale. As a solution you might treat each trap as a replicate in your analysis, but you would then introduce pseudo-replication because samples from a given meadow are inter-dependent through ecological interactions between the plots with traps (Hurlbert 1984). Likewise, in a laboratory experiment with two cages (control and treatment), you might consider each individual in a cage a replicate, but you would have difficulty to convince others that it was not some characteristic of the cage that produced the result. Another example is to use spatially clumped treatments: here, clusters of meadows with similar treatment. Now, underlying environmental gradients or local conditions could drive the result, not necessarily the fertilizer addition. Similarly, you should not compare moist Dutch meadows with dry Belgian meadows if your aim is to study the effect of moisture on carabids. The only exceptions for not properly replicating treatments concern studies on exceptionally rare (or dangerous) taxa, habitat types or phenomena.

### Suggestions for further research

Carabidologists have much to contribute to indicator studies. First of all, the researcher must adopt the conservationists’ view on what is an indicator. Second, the research must be properly carried out (see "Indicator hunt: common sense revisited"). Third, if the results suggest that carabids reliably reflect variation of high conservation relevance, the researcher should describe (i) the variables of the assemblage that best reflect this variation, (ii) the study conditions (context), (iii) the precision and accuracy of carabids in reflecting this variation based on, e.g., percent overlap, peak difference and confidence intervals, and (iv) the species or conditions that could not be easily observed without using carabids. Fourth, as the carabid ecological literature is vast (see "Carabids as model organisms"), and to increase the power of analyses, carabidologists should move on from two-tailed null hypothesis testing toward routinely formulating explicit, directional hypotheses − not just in indicator research but in modeling biological phenomena in general.

The various indicator categories ("Evaluation of carabids as indicators") provide potential for developing powerful management and conservation tools. Taxon, pollution, environmental and management indicators might be found by moving on from applying total richness toward using single-species abundances or their morphological/genetic variation, groups of specialists, functional groups, or structural characteristics of assemblages (as reflected by, e.g., affinity indices; Magura et al. 2006a; Déri et al. 2010). A different way to approach the indicator issue might be to study if the presence of certain species would indicate the *lack* of conservation values at a given site (‘negative indicators’). Keystone indicators, on the other hand, might be found through experiments with multiple trophic levels and manipulated abundances of potential competitors.

Early warning indicators are trendy because of their potential in assessing large-scale environmental alterations, but the concept could also be examined through ecological interactions and at smaller spatial scales. For example, responses of carabids to changes in combinations of temperature, soil chemistry and/or expansion of urban areas may be fruitful (see Knowlton and Graham 2010). The micro scale appears equally promising: carabids are physiologically extremely sensitive to sugars, salts, amino acids, pH and temperature (Merivee et al. 2004, 2005, 2008; Must et al. 2006). Thus, physiological alterations due to changes in these factors might function as early warning signals of currently minor environmental variation, such that cannot be observed by visual inspection of the environment. Some of these aspects could also be explored using affinity indices.

### Conclusions

No two species can precisely reflect each other, and one must be prepared for uncertainty and error when using an indicator. The competitive exclusion principle (Hardin 1960) postulates that members of a guild must be ecologically at least slightly different from one another to co-occur in terms of e.g. population dynamics, habitat and foraging requirements, aspects of reproduction and environmental grain size. Defining acceptable imprecision is a political question, but research can only determine confidence limits.

Indicators are assessment tools intended to be used in situations when habitats and species are lost, or conditions altered. Because humans will continue to utilizing the environment, some decrease in habitat area and, at some locations, quality is inevitable: biology competes with economics and social issues in policy. Detecting areas or sites of high conservation value assists in defining conservation priorities. Still, the conservationist may have to ask whether her/his statistically significant result is biologically or economically important, or whether a non-significant result is irrelevant. For example, if threatened or rare species are involved, the precautionary principle should apply (e.g., Haag and Kaupenjohann 2001): if a particular environmental impact is under evaluation, statistical non-significance should not be considered equal to no effect or zero difference (McGarvey 2007), and an indicator should be allowed to provide occasional ‘false positives’. The latter is important in protecting metapopulations, with both occupied and presently unoccupied habitat patches being necessary for the long-term persistence of an organism (Hanski 1999). Likewise, within a given area, local populations of carabids may differ in their reproductive capacity and other qualities, and consequently fluctuate partly independently (e.g., den Boer 2002).

To be useful in conservation, an indicator must have high and consistent predictive power that relates to particular conditions and/or rare species. We still lack the first clear-cut case showing carabids to reliably predict entities of high conservation and management interest. To fill this gap, (a) knowledge on the relationship between carabids and other taxa must be greatly increased, and (b) strict tests must be applied to evaluate indicator functioning as outlined above. We should soon be able to define a ‘niche’ for carabids in environmental assessments. Cases of carabids fulfilling criteria to be useful indicators will possibly be documented in the near future, but the indicator functioning of particular taxa may always remain context specific.
